# A RING to rule them all? Insights into the Map3k1 PHD motif provide a new mechanistic understanding into the diverse roles of *Map3k1*

**DOI:** 10.1038/cdd.2014.239

**Published:** 2015-01-23

**Authors:** T Suddason, E Gallagher

**Affiliations:** 1 Department of Medicine, Imperial College London, Du Cane Road, London, UK

## Abstract

Despite the sizable number of components that comprise Mapk cascades, Map3k1 is the only element that contains both a kinase domain and a plant homeodomain (PHD) motif, allowing Map3k1 to regulate the protein phosphorylation and ubiquitin proteasome systems. As such, Map3k1 has complex roles in the regulation of cell death, survival, migration and differentiation. Numerous mouse and human genetic analyses have demonstrated that *Map3k1* is of critical importance for the immune system, cardiac tissue, testis, wound healing, tumorigenesis and cancer. Recent gene knockin of *Map3k1* to mutate the E2 binding site within the Map3k1 PHD motif and high throughput ubiquitin protein array screening for Map3k1 PHD motif substrates provide critical novel insights into Map3k1 PHD motif signal transduction and bring a brand-new understanding to Map3k1 signaling in mammalian biology.

## Facts

- Of the 19 Map3ks only Map3k1 contains a plant homeodomain (PHD) motif, and is an E3 ubiquitin (Ub) ligase.
- The Map3k1 PHD motif regulates both Mapk cascade protein stability following hyperosmotic stress and Mapk pathway activation from transforming growth factor-β (Tgf-*β*) and epidermal growth factor (Egf) cytokine receptors by the Ub-proteasome system.
- The Map3k1 PHD motif is critical for stem cell differentiation, tumorigenesis, B-cell development, T-cell signaling, protecting cardiac tissue from damage and testis development.

## Open Questions

- Are divergent roles for the Map3k1 kinase domain and PHD motifs present within human breast cancers?
- Does the Map3k1 PHD motif regulate Tabs by non-canonical ubiquitination following CD40, and other Tnfrs, signal transduction?
- Are novel Map3k1 PHD motif substrates targeted by the Ub-proteasome system during apoptosis?

## Discovery and Early Characterization

**Figure 1:**
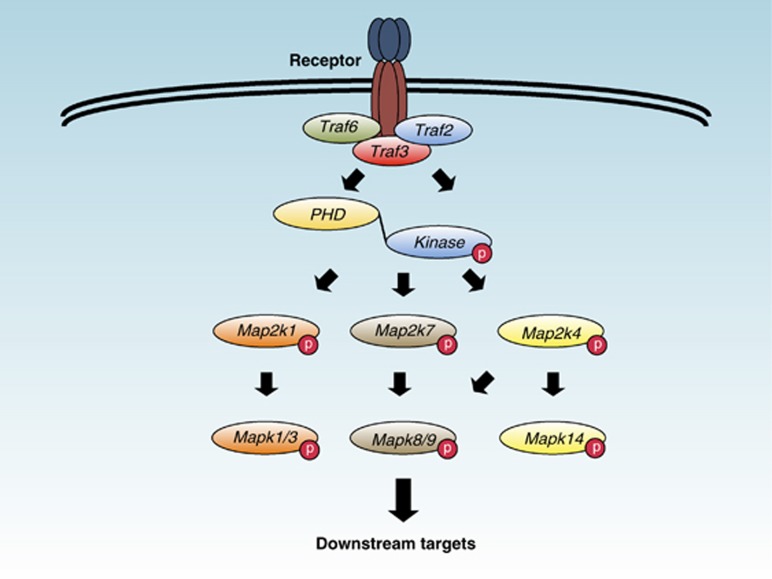
Schematic illustration of our early understanding of Map3k1 signal transduction. The Map3k1 kinase domain can phosphorylate and activate Map2ks that in turn phosphorylate and activate Mapks. Activated Mapk then phosphorylates downstream signaling targets in cells.

Mitogen-activated protein kinase (Mapk) kinase (Map2k) kinases (Map3ks) activate Mapks by the binding and phosphorylation of Map2ks (Figure 1).^1, 2, 3, 4^ Map3k1 (encoded by *Map3k1*, and also known as MEKK1) was initially partially cloned as a complimentary DNA (cDNA) that encoded the C-terminal 672 amino acid residues of the Map3k and contains the kinase domain.^5^ Notably, the Map3k1 kinase domain shows a significant sequence homology to the *Schizosaccharomyces pombe* kinase Byr2 and the *Saccharomyces cerevisiae* kinase Ste11, both Map3ks of the yeast pheromone response pathway.^5, 6^ But, despite its relatively high sequence similarity, Map3k1 cannot replace the function of Ste11 in yeast.^6^ Map3k1 is a serine and threonine kinase and a phospho-protein that was the second mammalian Map3k, after c-Raf, demonstrated to phosphorylate Map2k1 (also known as MEK1) within its activation domain.^7, 8, 9^ Subsequently, Map3k1 was shown to bind and activate Map2k4 (also known as MKK4 or JNKK1) that, in turn, phosphorylates the c-Jun N-terminal kinase (JNK) Mapk8 (also known as JNK1), Mapk9 (also known as JNK2) and Mapk14 (also known as p38-*α*).^10, 11, 12, 13^ Early work showed that both Tumor necrosis factor (Tnf) -*α*^14^ and crosslinking of the Tnf receptor (Tnfr) family member CD40 with antibodies activate Map3k1 in cell lines.^15^

The later cloning of the full-length rat Map3k1 cDNA revealed that Map3k1 possessed, in addition to its Ste11-like C-terminal kinase domain, a large N-terminal regulatory region.^16^ Overexpression of full-length Map3k1 activates Mapk1/3, Mapk8/9 and Mapk14 pathways in fibroblast cell lines.^1, 16^ Transfection of Map3k1 encoding cDNA into NIH3T3 cells leads to the activation of the nuclear factor *κ*-light-chain-enhancer of activated B-cell (NF-*κ*B) pathway.^17^ Under these circumstances Map3k1 can form a complex with and induce the phosphorylation of I*κ*B*α* kinases (Ikks) to activate the NF-*κ*B pathway in cell lines.^18^ By similar bioinformatics methods that identified the Map3k1 kinase domain, other functional protein motifs have been identified within Map3k1 through computer sequence alignment techniques, including the PHD,^19^ Ub interacting motifs^19^ and SWI2/SNF2 and MuDR domain.^20^ A multitude of Map3k1 binding partners have been found to date by a wide variety of molecular approaches (Table 1).

## Is Map3k1 a Mediator of Cell Death, Survival or Both?

Map3k1 was initially suggested to be a pro-apoptotic kinase after several failed attempts to create stable cell lines that overexpress this Map3k.^21^ Indeed, inducible expression of the Map3k1 kinase domain can sensitize Swiss 3T3 cells to UV-irradiation-induced cell death.^21^ Similarly, inducible expression of the Map3k1 kinase domain in L929 fibrosarcoma cells can increase their susceptibility to Tnf-*α*-induced apoptosis.^22^ Sequence analysis of full-length Map3k1 identified a short Cysteine-aspartic acid protease (Caspase) -3 cleavage site (^871^DTVD^874^), and its mutagenesis can prevent Map3k1-induced apoptosis caused by the overexpression of full-length Map3k1 encoding cDNA in cells.^22^ Caspase-3 cleavage at this site generates two Map3k1 fragments, the N-terminal fragment containing the PHD motif and the C-terminal fragment containing the kinase domain.^5, 19^ Expression of Map3k1 with a mutant Capase-3 cleavage site can also prevent Map3k1 cleavage in response to genotoxic stress in fibroblast cell lines and following CD95 (also known as Fas) -mediated apoptosis in the Jurkat T-cell line.^23, 24^ Anoikis, apoptosis induced by cellular detachment from extracellular matrices, can also initiate Map3k1 cleavage by Caspase-3, and an inactive kinase domain form of Map3k1 can inhibit anoikis-induced programmed cell death upon overexpression in Madin-Darby canine kidney cells.^25^ By contrast, Map3k1 is not susceptible to Caspase-3 cleavage following Madin-Darby canine kidney cell death induced by microtubule disruption drug treatment,^26^ demonstrating that Caspase-3 cleavage is not the only mechanism of Map3k1-mediated cell death. Expression of the anti-apoptotic protein B-cell lymphoma 2 can block Map3k1-mediated apoptosis, though B-cell lymphoma 2 overexpression by itself is insufficient to prevent the Map3k1 cleavage.^26^ Overexpression of Map3k1 initiates a substantial Mapk8/9 activation in many cell types and is the likely mechanism for the pro-apoptotic role of the Map3k1 kinase domain.^10, 13^ However, the induction of apoptosis by Mapk8/9 is a complicated process in cells and numerous mechanisms have been proposed to account for it, including activation of Itch by Mapk8 phosphorylation and Lys48-linked poly-Ub transfer onto cellular FLICE (FADD-like IL-1*β*-converting enzyme)-inhibitory protein (c-Flip) leading to c-Flip proteasomal degradation and the promotion of apoptosis,^27^ the control of p53 stability and cell death by Mapk8/9 phosphorylation of p53 at Ser6,^28^ and Mapk8/9 phosphorylation and regulation of B-cell lymphoma 2 family members to promote cell death.^4^

While the early analysis of Map3k1 by transfection and overexpression of its cDNA into cell lines suggested a pro-apoptotic role, these initial results in cell lines were then complicated by the contradictory finding that null mutant *Map3k1* (*Map3k1*^*−/−*^) mouse embryonic stem (ES) cells display enhanced cell death in response to hyperosmotic stress, low temperature shock and microtubule disruption drug treatment.^29, 30^ These important results demonstrated that Map3k1 has a more critical role in protecting mammalian cells from many types of cell death, and that the role of *Map3k1* in promoting apoptosis may well be a labyrinthine one. Similarly, *Map3k1*^*−/−*^ ES cell-derived cardiac myocytes show enhanced cell death in response to oxidative stress.^31^ Most likely Map3k1-dependent Mapk activation reduces cell death by the activation of pro-survival targets.^1, 3^

In addition to the Map3k1 kinase domain, roles for the Map3k1 PHD motif in cell death have been described.^19, 32, 33^ The Map3k1 PHD can mediate the transfer of Lys48-linked poly-Ub onto Mapk1, leading to the subsequent proteasomal degradation of Mapk1 in cell lines undergoing hyperosmotic stress-induced apoptosis.^19, 34^ Similarly, the Map3k1 PHD motif has been reported to mediate the transfer of Lys48-linked poly-Ub onto the c-Jun transcription factor to promote its degradation by the proteasome in *Map3k1*^*−/−*^ mouse embryonic fibroblast (MEF) cells undergoing hyperosmotic stress-induced apoptosis.^32^ The Map3k1 PHD may also act as E3 Ub ligase for c-Jun in cells undergoing cisplatin-induced apoptosis.^35^ Both the Map3k1 PHD and kinase domains are essential for microtubule disruption drug-induced Mapk8/9 activation and apoptosis in *Map3k1*^*−/−*^ DT40 cells.^36^

Recently, MarvelD3, a transmembrane component of tight junctions that is required for epithelial monolayer integrity during hyperosmotic stress, has been identified as a protein that forms a complex with Map3k1 in cells.^37^ MarvelD3 can relocalize Map3k1 in response to hyperosmotic stress and by this means can regulate Mapk8/9 activation.^37^ MarvelD3-mediated attenuation of Map3k1 signaling is critical for epithelial cell survival while undergoing hyperosmotic stress.^37^

## Cell Migration and Wound Healing

The generation of kinase-deficient Map3k1 (encoded by *Map3k1*^*ΔKD*^) expressing ES cells revealed that Map3k1 is critical for both serum- and lysophosphatidic acid (LPA) -induced Mapk8/9 phosphorylation.^38^ *Map3k1*^*ΔKD*^ ES cells also display reduced serum-induced migration in the Boyden chamber chemotaxis assays.^38^ Epidermal keratinocytes extracted from *Map3k1*^*ΔKD*^ mice have defective Tgf-*β*, Activin A- and Activin B-induced migration in cell culture plate-based wound healing assays.^2, 39^ *Map3k1*^*ΔKD*^ keratinocytes display reduced Mapk8/9 phosphorylation following treatment with Tgf-*β*, Activin A or Activin B.^39^ The molecular basis underpinning defective Map3k1-dependent migration during wound healing may be the reduced formation of actin stress fibers in Activin B-treated *Map3k1*^*ΔKD*^ keratinocytes.^39^ The formation of Activin B-induced actin stress fibers in keratinocytes is dependent upon Mapk8/9 activity because they can be ablated by the pre-treatment of keratinocytes with the SP600125 inhibitor compound.^2, 39^

*Map3k1*^*−/−*^ MEF cells are defective in their adherence to cell culture plates when centrifuged at low speed.^40^ Like *Map3k1*^*ΔKD*^ ES cells, *Map3k1*^*−/−*^ MEF cells display significantly reduced migration toward serum in the transwell migration assays.^38, 40^ Similarly, migration toward fibronectin or fibronectin and Egf is reduced in *Map3k1*^*−/−*^ MEF cells.^40^ Map3k1 has been shown by two groups to localizes to focal adhesions in fibroblasts,^40, 41^ and less Vinculin, a critical cytoskeletal protein found in focal adhesions, is detected at the focal adhesions of *Map3k1*^*−/−*^ MEF cells.^40^ Egf treatment of MEF cells leads to the formation of a complex between focal adhesion kinase (Fak) and Map3k1.^40^ *Map3k1*^*−/−*^ MEF cells also display both reduced Mapk1/3 phosphorylation in response to Egf or Fibroblast growth factor-2 treatment and decreased Calpain activation, a calcium-dependent cysteine protease that is activated by Mapk1/3 phosphorylation.^40, 42^

## Lymphocyte Differentiation and Effector Responses

Naïve CD4^+^ T cells purified from the secondary lymphoid tissues of *Map3k1*^*ΔKD*^ mice and cultured under T helper (Th) 2 polarizing conditions secrete enhanced levels of Interleukins 4, 5, 10 and 13.^43^ By contrast, Th1 differentiation proceeds normally for CD4^+^ T cells isolated from *Map3k1*^*ΔKD*^ mice.^43^ The aberrant Th2 phenotype identified in CD4^+^ T cells derived from *Map3k1*^*ΔKD*^ mice resembles the overproduction of Th2 cytokines found in *Itchy* mice, that harbor a promoter rearrangement mutation that ablates the expression of homologous to the E6-AP carboxyl terminus (HECT) E3 Ub ligase Itch.^44, 45^ Overproduction of Th2 cytokines is also a phenotype of CD4^+^ T cells isolated from *Mapk8*^*−/−*^ mice and transgenic mice engineered to overproduce JunB.^46, 47^ *Map3k1*^*ΔKD*^, *Mapk8*^*−/−*^ and *Itchy* Th2 cells all produce similarly deficient responses in a mouse T-cell allergy model.^48^ As well as having an important role in CD4^+^ T-cell differentiation the Map3k1 kinase domain also has a negative regulatory role in the proliferative-expansion of CD8^+^ T cells.^49^

Either Map3k1 or Mapk8-Map2k7 fusion protein can enhance Itch E3 Ub ligase activity toward its substrate JunB in HEK 293 cells.^43, 45^ After T-cell receptor engagement Map3k1-dependent Mapk8 signaling is activated in T cells and Itch undergoes Mapk8-mediated phosphorylation of Ser199, Ser232 and Thr222 within the Itch Pro-rich region.^50^ Direct Itch phosphorylation by Mapk8 disrupts an inhibitory interaction that occurs between the Itch WW domains, that mediate protein–protein interactions, and the HECT domain, the E3 Ub ligase, and this change in Itch conformation leads to the significantly increased activity of the HECT domain.^50^ Map3k1 and Itch also form a complex within T cells as Itch is activated by Mapk8 phosphorylation.^51^

Despite *Map3k1*^*ΔKD*^ T cells displaying skewed Th2 cytokine production, *Map3k1*^*ΔKD*^ mice show both significantly reduced germinal center formation within their secondary lymphoid tissues and production of antibodies in response to thymus-dependent, but not thymus-independent, antigens.^52, 53, 54^ As suggested by early work measuring Map3k1 activation in B-cell lines following CD40 engagement with antibodies, Map3k1 was found to be necessary for CD40 ligand (CD40L, also known as CD154) -mediated activation of Mapk8/9 and Mapk14 in B cells.^15, 52, 54^ *Map3k1*^*ΔKD*^ B cells have significantly reduced c-Jun phosphorylation and defective expression of both Cyclin D2, a regulator of cyclin-dependent kinases, and Activation-induced deaminase, a protein important in antibody diversity, that likely explains the poor humoral immune responses seen in *Map3k1*^*ΔKD*^ mice.^52, 54^

Following the engagement of CD40 by CD40L, and also many other Tnfrs by their ligands, Tnf receptor-associated factor (Traf) 2 (Traf2), Traf3, Ub-conjugating enzyme E2 N (Ube2N, also known as Ubc13), cellular inhibitor of apoptosis proteins 1 and 2 (c-Iap1/2), Ikk*γ* and Map3k1 are recruited rapidly to the CD40 receptor.^52, 53^ Traf2, Ube2N and Ikk*γ* are all necessary components for both the assembly of the signal transduction complex at the receptor and the activation of Map3k1 and its downstream Mapks.^52, 53^ The CD40 Mapk signaling complex is inactive at the receptor, but the complex then translocates from the CD40 receptor into the cytosol to become active after the transfer of Lys48-linked poly-Ub by c-Iap1/2 onto Traf3 and the subsequent degradation of Traf3 by the proteasome.^52, 53^

## Role in Stem Cells and Cardiac Myocytes

Initial analyses using *Map3k1*^*−/−*^ ES cells showed they are deficient in Mapk activation in response to a wide variety of stimuli, including microtubule disrupting drugs, low temperature shock, hyperosmotic stress and growth factors present within the serum.^29, 30^ As stated above, *Map3k1*^*−/−*^ ES cells have a greater propensity to enter apoptosis following hyperosmotic stress or treatment with microtubule disrupting drugs than wild-type (WT) ES cells.^29, 30^ *Map3k1*^*ΔKD*^ ES cells display reduced Mapk8/9 activation in response to pro-inflammatory agonists, serum and LPA.^38^ *Map3k1*^*−/−*^ ES cell-derived cardiac myocytes display increased sensitivity to apoptosis following hydrogen peroxide-induced stress. In response to hydrogen peroxide-induced stress *Map3k1*^*−/−*^ ES cell-derived cardiac myocytes show reduced Mapk8/9 phosphorylation.^31^

Map3k1 can be activated by heart-restricted expression of G*α*q, a heterotrimeric G protein subunit, in transgenic mice.^55^ *Map3k1*^*−/−*^ ES cell-derived cardiac myocytes have reduced Mapk8/9 phosphorylation following treatment with the *α*_1_-Adrenergic receptor agonist phenylephrine.^55^ *Map3k1*^*−/−*^ mice display increased cardiac mass, larger cardiac myocytes, elevated Atrial natriuretic factor (Anf) expression, impaired Mapk8/9 phosphorylation by G*α*q and improved ventricular function.^55^ *Map3k1*^*−/−*^ mice also show reduced Mapk8/9 phosphorylation following transverse aortic constriction, an indicator that Map3k1 may regulate Mapk8/9 activation following cardiac pressure overload, where cardiac muscle is forced to contract while undergoing excessive afterload.^56^ Pressure overload causes significant cardiac hypertrophy and increased expression of Anf in *Map3k1*^*−/−*^ mice, which also show higher mortality and a greater ratio of lung to body mass.^56^ Map3k1 may be required for pressure overload-induced Mapk8/9 activation and enhanced production of Tnf-*α* and Tgf-*β* cytokines.^56^ Map3k1 can function in the cardiovascular system to promote cardiac myocyte cell survival, reduce inflammation and protect against cardiac failure.^56^

## Roles in Cancer

Early analyses identified a potential role for MAP3K1 in Androgen receptor signaling in prostate cancer cell lines.^57^ The Androgen receptor-positive LNCaP cell line, derived from Androgen-sensitive human prostate adenocarcinoma cells, undergoes apoptosis when transduced with a retroviral vector that overexpresses the Map3k1 kinase domain.^57^ By contrast, Androgen receptor-negative DU145 cells and PC3 cells do not enter apoptosis when transduced with retrovirus expressing the Map3k1 kinase domain.^57^ Co-transduction of the Androgen receptor and Map3k1 kinase domain into DU145 cells leads to apoptosis.^57^ Conversely, transfection of kinase-inactive Map3k1 expressing cDNA into BxPC-3, PANC-1, MIAPaCa-2 and AsPC-1 pancreatic cancer cell lines promoted cell death in pancreatic cancer cell lines, suggesting a role for Map3k1 kinase domain signaling in tumor cell survival.^58^

Small interfering RNA knockdown of MAP3K1 expression in the invasive human adenocarcinoma cell line MDA-MB-231, that contains an aberrant *Wnt7b* oncogene, causes a significant reduction in urokinase-type plasminogen activator (uPA), a serine protease whose expression can correlate with tumor malignancy, activity.^59, 60^ Knockdown of MAP3K1 in MDA-MB-231 cells causes reduced migration towards serum growth factors in transwell migration assays when compared with MDA-MB-231 cells transfected with a control small interfering RNA.^60^ These results suggest a potential role for MAP3K1 in regulating both tumor malignancy and cancer cell invasion into normal tissues.

*Map3k1*^*−/−*^ mice crossed to a polyoma virus middle T antigen (PyMT) transgene under control of the mouse mammary tumor virus long terminal repeat (MMTV LTR) show significantly delayed development of lung metastases.^60^ The dissemination of cancerous cells from *Map3k1*^*−/−*^ mammary tumors is reduced, though the eventual formation of lung metastases still occurs in mice.^60^ The delay in the development of lung metastasis observed in *Map3k1*^*−/−*^ mice is perhaps caused by the reduced integrity of the basement membranes surrounding the *Map3k1*^*−/−*^ mammary tumors.^60^ Map3k1 may regulate both proteolytic degradation and migration of tumor cells in mice.^60^

Screening for breast cancer susceptibility alleles has identified, amongst several genes, *MAP3K1* as a causative gene for breast cancer.^61^ In fact, ~12% of all luminal A breast cancer tumors contain mutations within *MAP3K1* or *MAP2K4*, kinases that can regulate MAPK8/9 and MAPK14 signaling.^62^ Almost all breast cancer *MAP3K1* and *MAP2K4* mutations are found within luminal A tumors, and *MAP3K1* and *MAP2K4* mutations are largely mutually exclusive of each other.^62^ Although the role for MAP3K1 in some forms of breast cancer is substantiated by numerous reports,^61, 63, 64, 65, 66, 67, 68^ the importance of MAP3K1 in other cancers is far less certain. A recent low-copy transposon mutagenesis screening methodology in mice identified *Map3k1* as a potentiator of melanoma.^69^ Point mutations within introns 9 and 10 of *Map3k1* produce truncated forms of Map3k1 that lack the N-terminal regulatory region.^69^ N-terminal truncation of Map3k1 leads to enhanced Mapk1/3 phosphorylation in melanoma tumors, and provides a possible mechanism to explain how Map3k1 may drive malignancy in melanoma.^69^ It is also notable that host immunodeficiency may also contribute to the prognosis of *Map3k1*-dependent cancers.^70^

## New Insights into Map3k1 PHD Motif Signaling by Gene Knockin of *Map3k1*

To better understand the functions of the PHD motif in mammalian biology we first modeled the Map3k1 PHD upon the known structure of the Deltex 2 Really Interesting New Gene (RING), which has homology with the Map3k1 PHD motif, and mutated conserved residues within the E2 binding region of the PHD structure (Cys438Ala and Ile440Ala, the Map3k1 mPHD mutation) to inactivate the E3 Ub ligase, and the Map3k1 mPHD mutation does not significantly reduce kinase domain activity.^34, 71, 72^ Transfection of Map3k1 mPHD expressing cDNA into HEK 293 cells revealed that the Map3k1 mPHD protein displays significantly impaired Map3k1 auto-ubiquitination.^19, 33, 72^ Analysis of the E2 conjugating enzymes that can act in concert with Ube1 and the Map3k1 PHD revealed that Ub-conjugating enzyme E2D (Ube2D) 2 (Ube2D2), Ube2D3 and Ube2N:Ub-conjugating enzyme E2 variant 1 (Ube2V1) can all mediate Map3k1 auto-ubiquitination.^72^ Previously it was known that the Map3k1 PHD may utilize Ube2D2 to transfer poly-Ub onto Mapk1.^19, 33^ Immunoblotting with anti-Lys63-linked Ub monoclonal antibodies demonstrated that the Map3k1 PHD motif largely forms Lys63-linked poly-Ub chains as opposed to linear poly-Ub chains.^72^ Lys63-linked poly-Ub is efficiently removed from Map3k1 by the deubiquitinating enzymes Ub-specific protease 2, 7 and 8.^72^

Mutation of *Map3k1* alleles to express the Map3k1 mPHD (encoded by *Map3k1*^*mPHD*^) revealed that *Map3k1*^*mPHD*^ ES cells do not exhibit defective Mapk1 expression following long-term hyperosmotic stress as has previously been suggested by the overexpression analysis in cell lines.^19, 33, 72^ This finding suggested that while Mapk1 is a substrate for Map3k1 PHD ubiquitination in cells, Map3k1 may not have a critical role in regulating Mapk1 by poly-Ub and there may also be other Mapk1 E3 Ub ligases that perform this function in cells undergoing hyperosmotic stress.^19, 33^ However, the kinetics of the full-length Map3k1 degradation in response to hyperosmotic stress are altered in *Map3k1*^*mPHD*^ ES cells.^72^ Our findings suggested that the Map3k1 PHD can critically regulate the rapidity of its own degradation while undergoing hyperosmotic stress by auto-ubiquitination, but that other E3 Ub ligases (e.g. Deltex family E3 Ub ligases) may also act as E3 Ub ligases toward Map3k1 following hyperosmotic stress in the absence of a functional PHD motif.^72, 73^ Surprisingly, we identified a defective Mapk activation in *Map3k1*^*mPHD*^ ES cells following their treatment with microtubule disrupting drugs and the cytokines Tgf-*β* or Egf, suggesting that the Map3k1 PHD, in fact, has an unexpected and critical role in regulating Mapk activation.^72^

Neither of the known Map3k1 PHD substrates (Mapk1 and c-Jun) provide an obvious explanation for the new critical role found for the PHD motif in cytokine-induced Mapk activation,^19, 32, 33, 61^ so we searched for novel substrates using a high throughput screening approach that analyzed a library of over 9400 human full-length proteins.^74, 75^ Our Ub protein array screening methodology identified 82 new proteins as potential substrates for a ubiquitination reaction containing Ube1, Ube2N:Ube2V1 and the Map3k1 PHD motif.^72^ Many of the PHD motif substrates are molecular scaffold proteins involved in signal transduction, and bioinformatics analysis suggested that Tgf-*β* activated kinase 1-binding protein (Tab) 1 (Tab1) was critical for Tgf-*β*-induced Mapk activation.^72, 76^

**Figure 2:**
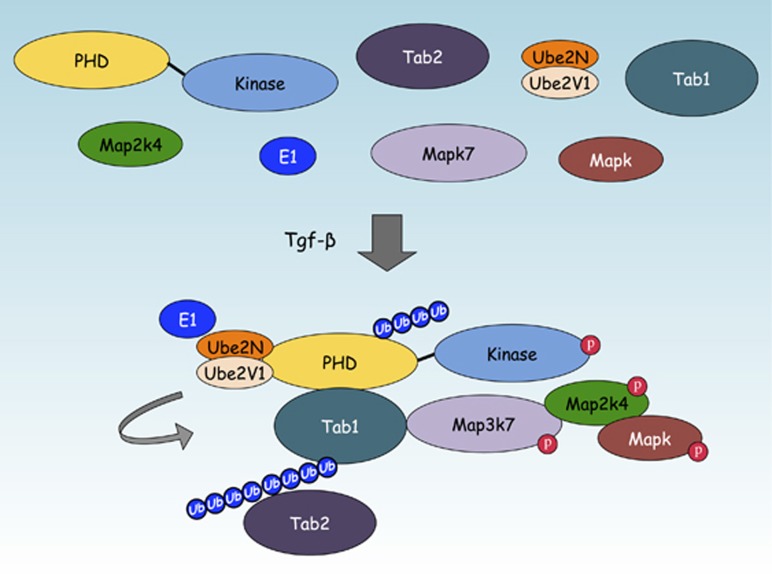
The Map3k1 PHD motif regulates Tabs in response to cytokine stimulation. Following Tgf-*β* treatment, the Map3k1 PHD motif binds and transfers Lys63-linked poly-Ub onto Tab1 to enhance Map3k7 activation. Tab2, although not a Map3k1 PHD motif substrate, can be recruited to the Map3k1:Map3k7 Ub signaling complex by the Ub binding ZnF motif of Tab2.

**Figure 3:**
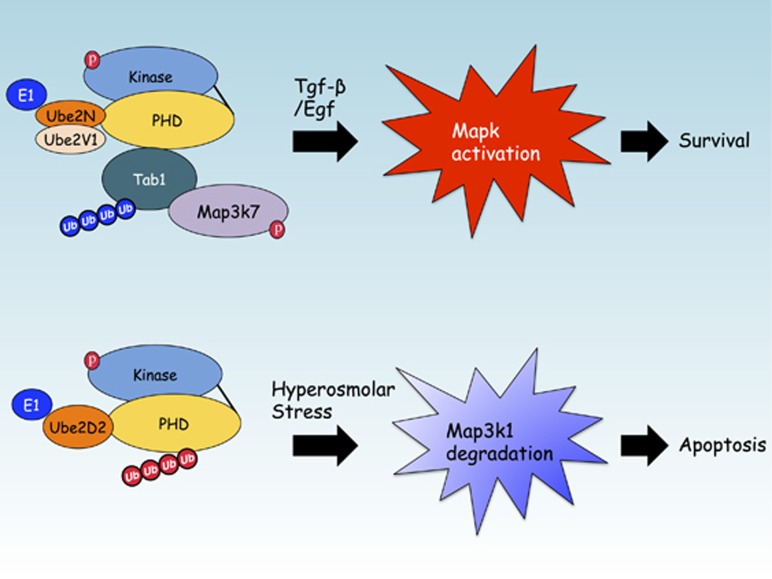
The signal transduction role for the Map3k1 PHD motif during cell survival or apoptosis. In response to Tgf-*β* or Egf cytokines Map3k1 ubiquitinates Tab1 with Lys63-linked poly-Ub to activate Map3k7 and Mapk. This promotes both the differentiation and survival of stem cells. In response to hyperosmotic stress, stem cells enter apoptosis and the Map3k1 PHD motif transfers Lys48-linked poly-Ub onto itself, in conjunction with other E3 Ub ligases, to potentiate the degradation of the Mapk signaling cascade by the proteasome.

Ubiquitination assays by orthogonal approaches confirmed that the Map3k1 PHD can transfer Lys63-linked poly-Ub onto recombinant Tab1, and also other scaffold proteins, namely Traf2, TNFAIP3 interacting protein (Tnip) 1 (Tnip1), Tnip2 and Signal transducing adapter molecule 1 (Stam1).^72^ Of these Map3k1 PHD motif substrates, and after immunoprecipitation from the WT and *Map3k1*^*mPHD*^ ES cells stimulated by Tgf-*β*, only Tab1 was found to be significantly ubiquitinated by Lys63-linked poly-Ub in WT and not *Map3k1*^*mPHD*^ ES cells.^72^ Generation of *Tab1*^*−/−*^ ES cells revealed that they, like *Map3k1*^*mPHD*^ ES cells, are deficient in Egf- and Tgf-*β*-induced Mapk and Map3k7 (also known as Tak1) activation.^72^ Mapping of the Tab1 ubiquitination sites mediated by the Map3k1 PHD and Ube2N:Ube2V1 identified Lys294, Lys319, Lys335 and Lys350 as being important for Tab1 ubiquitination by the PHD motif. Map3k1 can interact with and transfer Lys63-linked poly-Ub onto Tab1 by its PHD motif to potentiate the protein–protein interaction between Tab1 and Map3k7.^72^ Tab2, though not itself a Map3k1 PHD substrate, can be recruited into the Tab1:Map3k1 Ub complex to form a ternary complex that is dependent upon the Tab2 zinc finger (ZnF), a motif that can interact with proteins that possess Lys63-linked poly-Ub chains.^72, 77^ Recruitment of Tab2 into the Map3k1:Tab1 signaling complex may facilitate a further downstream signaling from Tgf-*β* receptors (Tgf*β*rs) and Egf receptors (Egfrs) (Figure 2). The formation of a Ub signaling complex between Tab1:Map3k1:Map3k7 offers a plausible explanation for why *Map3k1*^*mPHD*^ ES cells or WT ES cells treated with the Map3k7 chemical inhibitor (5Z)-7-oxozeaenol both lose Mapk activation following stimulation by Tgf-*β* or Egf cytokines.^72^ Chemical inhibition using the Ube2N inhibitor NSC697923 demonstrates that Ube2N is also critical for Map3k7 and Mapk activation in ES cells following treatment with Tgf-*β* or Egf, and Ube2N is also important for Tgf-*β*-induced Mapk activation in breast cancer cells.^72, 78^ However, unlike CD40 signaling in B cells, where Traf2 is critical for Mapk8/9 and Mapk14 activation,^52, 53^ instead for Tgf*β*rs Traf6 is critical for Mapk activation.^79^ The identification of a new PHD motif substrate that forms the lynchpin between Tgf-*β*-dependent Mapk pathway activation and stem cell differentiation further complicates the role for the Map3k1 PHD motif in apoptosis, and suggests that under conditions of cell death induced by hyperosmotic stress Map3k1 may silence Mapk signaling, while when stimulated by cytokines the Map3k1 PHD plays a role in promoting cell survival and differentiation by activating Mapks (Figure 3).

Both Tab1 and the Map3k1 PHD can negatively regulate neuroectoderm genes and enhance long-term expression of mesoderm genes in ES cells as they differentiate from pluripotent stem cells into embryoid bodies in cell culture.^72^ *Mapk8*^*−/−*^*/Mapk9*^*−/−*^ double deficiency in ES cells has demonstrated that Mapk8/9 are important for driving ES cell differentiation.^80^ *Mapk14* is also known to have an important role in ES cell differentiation by regulating neuroectoderm and mesoderm formation.^81, 82^ Despite these results no compound mutant *Mapk8*^*−/−*^*/Mapk9*^*−/−*^- and *Mapk14*^*−/−*^-deficient ES cells have been generated and analyzed to date that would produce the similar differentiation defects observed in *Map3k1*^*mPHD*^ or *Tab1*^*−/−*^ ES cells. Transplantation of immunodeficient host mice with either *Tab1*^*−/−*^ or *Map3k1*^*mPHD*^ ES cells causes aberrant tumors to form, and these have altered tissue composition and are of smaller mass and size.^72^ Add-back of Tab1, but not lysine-mutated (Lys294Ala, Lys319Ala, Lys335Ala and Lys350Ala) Tab1, into *Tab1*^*−/−*^ ES cells restores normal ES cell differentiation and tumorigenesis, demonstrating that the lysines in Tab1 ubiquitinated by the Map3k1 PHD motif are critically important for ES cell differentiation and tumor formation in mice.^72^

Analysis of *Map3k1*^*mPHD*^ knockin mice is complicated by their early lethality during embryogenesis, a more severe phenotype than the partial lethality observed in *Map3k1*^*ΔKD*^ mice.^72, 83^ Indeed, the combination of aberrant regulation of the Ub-proteasome system and defects in Mapk signaling provide a plausible explanation for the more severe phenotypes of *Map3k1*^*mPHD*^ mice.^19, 33^ However, mature *Map3k1*^*mPHD/+*^ mice are viable for phenotypic analysis and have demonstrated that signal transduction by the Map3k1 PHD motif is critical for B-cell development beyond the Pro-B-cell stage, T-cell receptor signal transduction and Itch phosphorylation within the pro-rich region , protecting cardiac tissue and maintaining the Leydig cell population within testis.^72^

## Summary

**Figure 4:**
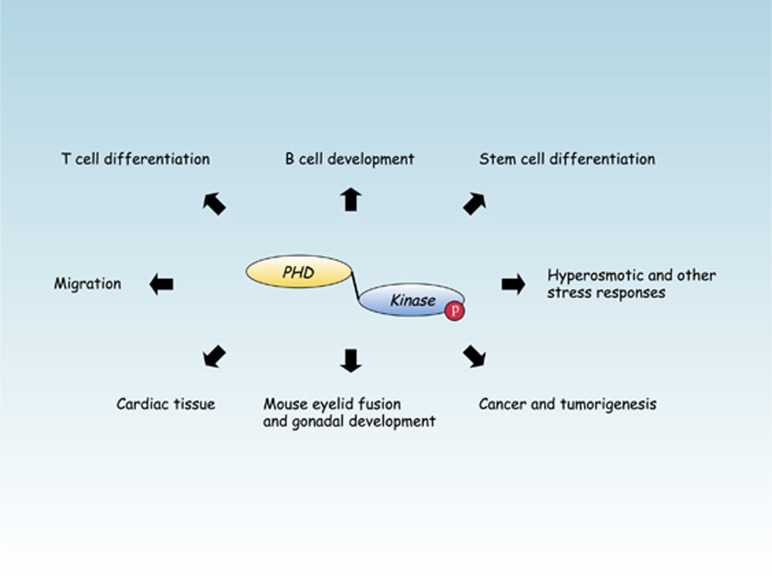
Tissues, cells and diseases where mouse or human genetics have demonstrated *Map3k1* signaling to be of importance.^2, 30, 45, 52, 99^

From its initial discovery as the second Map2k1 kinase to defining the role of Map3k1 signal transduction in tumorigenesis and breast cancer, the road to understanding the intricate mechanisms of Map3k1 signaling has seemed to go on forever (Figure 4). Our new insights into the role of the Map3k1 PHD motif provide a fresh perspective into how *Map3k1* signaling can regulate both the Ub-proteasome and protein phosphorylation systems. Analysis of Map3k1 domain-specific signaling has now revealed several important brand-new insights. At cytokine receptors and in response to microtubule disruption the Map3k1 PHD motif and kinase domain are both required for Mapk activation. But, while the Map3k1 kinase domain is required for hyperosmotic stress-dependent Mapk activation, the PHD motif is dispensable for Mapk activation under this circumstance, and instead enhances the kinetics of full-length Map3k1 degradation. Analysis of the Map3k1 PHD by mouse genetics has also demonstrated that, like the Map3k1 kinase domain, the PHD motif is important for lymphocyte T-cell receptor signaling, cardiac tissue damage and stem cells. However, it is notable that disruption of the Map3k1 PHD motif has a more dramatic effect upon B-cell development than kinase domain ablation, the degree of embryonic lethality encountered is more severe in *Map3k1*^*mPHD*^ than *Map3k1*^*ΔKD*^ mice and there are more severe mesoderm and neuroectoderm differentiation defects in *Map3k1*^*mPHD*^ than *Map3k1*^*ΔKD*^ stem cells. As such, modulation of the Map3k1 PHD motif may eventually provide an attractive alternative target to the kinase domain for future drug discovery.^84^

## Figures and Tables

**Table 1:** Listing of Map3k1 binding partners

**Protein**	**Function**	**Reference**
Nck interacting kinase	Kinase	^85^
14-3-3	Scaffold	^86^
Mapk9	Kinase	^87^
*α*-actinin	Microfilament protein	^41^
c-Raf	Kinase	^88^
Mapk1	Kinase	^88^
RhoA	Small GTPase	^89^
Cdc42	Small GTPase	^90^
Ras	Small GTPase	^91^
Rac	Small GTPase	^90^
p115 RhoGAP	GTPase-activating protein	^92^
Map2k4	Kinase	^12^
Map2k7	Kinase	^12^
Map2k1	Kinase	^5^
c-Jun	Transcription factor	^32^
Itch	HECT E3 Ub ligase	^51^
Traf2	Scaffold	^93^
Grb2	Adapter	^94^
Axin	Scaffold	^95^
Fak	Kinase	^96^
Deltex 1	E3 Ub ligase	^73^
Ikk*α*	Kinase	^18^
Ikk*β*	Kinase	^18^
Ikk*γ*	Scaffold	^53^
Tax	Nuclear factor	^97^
Han11	Scaffold	^98^
MarvelD3	Tight junction protein	^37^
Tab1	Scaffold	^72^

## Abbreviations

Anf: Atrial natriuretic factor
Bcl-2: B-cell lymphoma 2
c-Iap1/2: Cellular inhibitor of apoptosis proteins 1 and 2
Caspase: Cysteine-aspartic acid protease
DUBs: deubiquitinating enzymes
Egf: Epidermal growth factor
Egfrs: Egf receptors
ES: embryonic stem
Fak: Focal adhesion kinase
c-Flip: FLICE (FADD-like IL-1*β*-converting enzyme)-inhibitory protein
HECT: Homologous to the E6-AP Carboxyl Terminus
Ikks: I*κ*B*α* kinases
LPA: lysophosphatidic acid
MDCK: Madin-Darby canine kidney
MMTV LTR: mammary tumor virus long terminal repeat
Map2k: Mapk kinase
Map3k: Map2k kinase
Mapk: Mitogen-activated protein kinase
MEF: mouse embryonic fibroblast
NF-*κ*B: Nuclear factor *κ*-light-chain-enhancer of activated B cells
PHD: plant homeodomain
PyMT: Polyoma Virus middle T antigen
PRR: Pro-rich region
RING: Really Interesting New Gene
Stam1: Signal transducing adapter molecule 1
SWIM: SWI2/SNF2 and MuDR
siRNA: Small interfering RNA
Tab: Tgf-*β* activated kinase 1-binding protein
Tgf*β*rs: Tgf-*β* receptors
Th: T helper
Tnip: TNFAIP3 interacting protein
Tnfr: Tnf receptor
Traf: Tnf receptor-associated factor
Tgf-*β*: Transforming growth factor-*β*
Tnf: Tumor necrosis factor
Ube2N: Ub-conjugating enzyme E2 N
UIMs: Ub-interacting motifs
Ub: Ubiquitin
Upa: urokinase-type Plasminogen Activator
Ube2D: Ub-conjugating enzyme E2D
Ube2V1: Ub-conjugating enzyme E2 variant 1
Usp: Ub-specific protease
ZnF: zinc finger
